# Supplementary material for: Serrulin: A Glycine-Rich Bioactive Peptide from the Hemolymph of the Yellow Tityus serrulatus Scorpion
Source: Toxins (Basel). 2019 Sep 6;11(9):517. doi: 10.3390/toxins11090517 (PMC6784228; doi:10.3390/toxins11090517)
Supplement: Supplementary file 1 [file toxins-11-00517-s001.pdf]

Supplementary Materials: Serrulin: A Glycine-Rich Bioactive Peptide from the Hemolymph of the Yellow *Tityus serrulatus* Scorpion

Thiago Oliveira, Ursula Castro de Oliveira and Pedro Ismael da Silva Junior

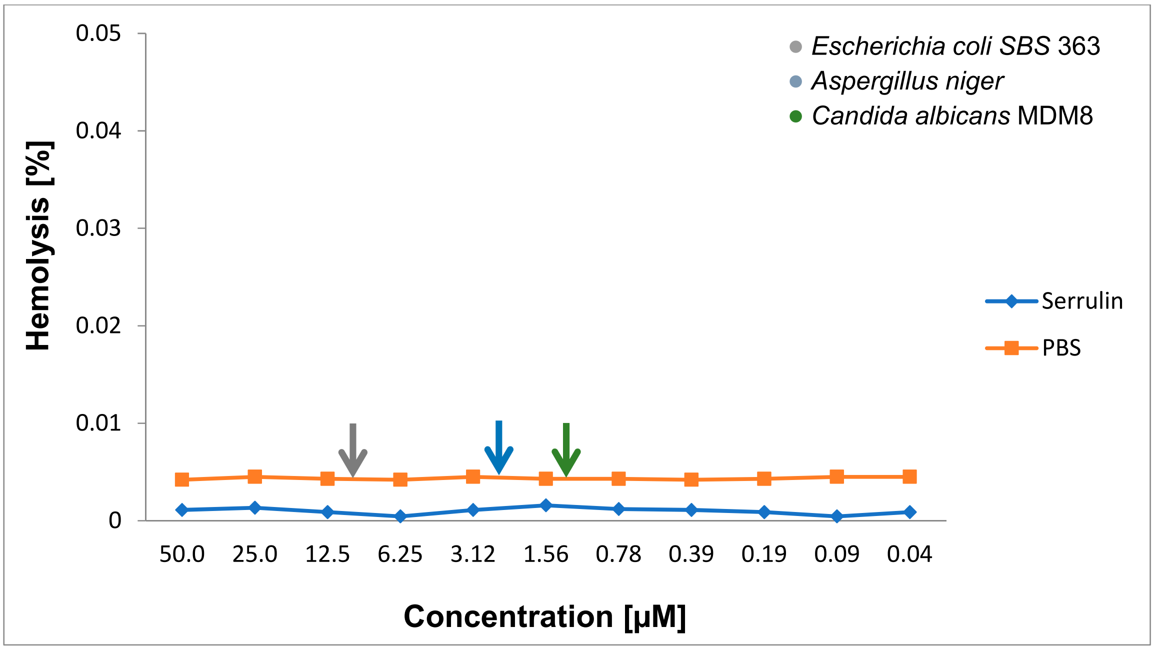

Figure S1. Hemolytic effects of serrulin on human erythrocytes. The concentration-response curve of the hemolytic activity of the peptide shows the maximum tested concentration (50 µM), which is very similar to that of the negative control (phosphate-buffered saline (PBS)).

```
Number of amino acids: 37

Molecular weight: 3048.29

Theoretical pI: 11.10

Amino acid composition: CSV format
Ala (A)   0       0.0%
Arg (R)   2       5.4%
Asn (N)   0       0.0%
Asp (D)   0       0.0%
Cys (C)   0       0.0%
Gln (Q)   0       0.0%
Glu (E)   0       0.0%
Gly (G)  26      70.3%
His (H)   0       0.0%
Ile (I)   2       5.4%
Leu (L)   0       0.0%
Lys (K)   2       5.4%
Met (M)   0       0.0%
Phe (F)   4      10.8%
Pro (P)   0       0.0%
Ser (S)   0       0.0%
Thr (T)   0       0.0%
Trp (W)   0       0.0%
Tyr (Y)   1       2.7%
Val (V)   0       0.0%
Pyl (O)   0       0.0%
Sec (U)   0       0.0%

 (B)   0          0.0%
 (Z)   0          0.0%
 (X)   0          0.0%


Total number of negatively charged residues (Asp + Glu): 0
Total number of positively charged residues (Arg + Lys): 4

Atomic composition:

Carbon      C          133
Hydrogen    H          195
Nitrogen    N           45
Oxygen      O           39
Sulfur      S            0

Formula: C133H195N45O39
Total number of atoms: 412

Extinction coefficients:

This protein does not contain any Trp residues. Experience shows that
this could result in more than 10% error in the computed extinction coefficient.

Extinction coefficients are in units of  M-1 cm-1, at 280 nm measured in water.

Ext. coefficient     1490
Abs 0.1% (=1 g/l)   0.489

Estimated half-life:

The N-terminal of the sequence considered is G (Gly).

The estimated half-life is: 30 hours (mammalian reticulocytes, in vitro).
                            >20 hours (yeast, in vivo).
                            >10 hours (Escherichia coli, in vivo).
```

Figure S2. Physico-chemical parameters of serrulin. Data obtained by the sequence analysis, "GFGGGRGGFGGGRGGFGGGGIGGGGFGGGYGGGKIKG," in ExPASy ProtParam. Information on the following is provided: The theoretical pI, molecular weight, total number of negatively and positively charged residues, atomic composition, extinction coefficients and percentage of amino acids found in the sample.

B7P914 B7P914_IXOSC - **Glycine-rich secreted protein, putative** Ixodes scapularis (Black-legged tick) (Deer tick)

E-value: 1.8e-12
Score: 161
Ident.: 68.1%
Positives : 72.3%
Query Length: 37
Match Length: 192

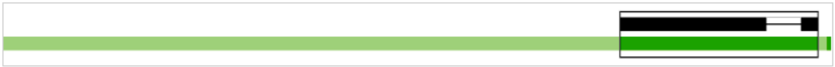

```
Query                  1    GFGGGRGGFGGGRGG-FGGGGIGGGGFGGGYGGG---------KIKG    37
                            G+GGG GGFGGG GG FGGGG GGGGFGGGYGGG         K+KG
B7P914 B7P914_IXOSC   144    GYGGG-GGFGGGLGGGFGGGGFGGGGFGGGYGGGHGGGGIAVIKVKG   189
```

Figure S3. Results obtained by searching with BLAST (Basic Local Alignment Search Tool) using Uniprot (Universal Protein resource). The amino acid sequence of serrulin had a similarity of 68% to a protein found in the tick transcriptome of the species, *Ixodes scapularis*.
